# Municipal solid waste gasification for resilient energy systems: advancing sustainable crisis preparedness in the wake of COVID-19

**DOI:** 10.3389/fbioe.2025.1691738

**Published:** 2025-11-07

**Authors:** Mohammad Rehan, Khurram Shahzad, Nadeem Ali, Abdul-Sattar Nizami

**Affiliations:** 1 Center of Excellence in Environmental Studies (CEES), King Abdulaziz University, Jeddah, Saudi Arabia; 2 Graduate School of Energy and Environment, Korea University, Seoul, Republic of Korea; 3 Sustainable Development Study Centre, Government College University, Lahore, Pakistan; 4 LL Liminal Lab Research Group, Architecture Department, Faculty of Architecture and Urbanism, UTE University, Quito, Ecuador

**Keywords:** municipal solid waste, life cycle assessment, techno-economic analysis, wasteto energy, syngas, gasification

## Abstract

The pandemic increased global waste problems, especially medical and MSW (Municipal Solid Waste), showing the urgent need for sustainability. This research evaluates the environmental and economic sustainability of transforming MSW into syngas and electricity via gasification, using life cycle assessment (LCA) and techno-economic analysis. This research simulates syngas production and energy recovery for a 50-ton-per-day gasification plant, with a focus on waste disposal in Jeddah, Saudi Arabia. The LCA findings reveal substantial overall reductions in environmental impacts, with a climate change benefit of −100 kg CO_2_ eq. per ton of waste processed, alongside offsets in fossil depletion (−32.5 kg oil eq.) and particulate matter formation (−0.025 kg PM2.5 eq.). Financially, the system is sound, generating $516,474 yearly, with a 4.8-year return and 208% return on investment (ROI). The research supports various Sustainable Development Goals (SDGs), including SDG 7, SDG 12, and SDG 13, while also addressing waste issues related to the COVID-19 pandemic. Pilot-scale validation is necessary to address real-world variability when relying on simulated data. The results support gasification as a robust, circular technology for enhancing general MSW management resilience, providing policymakers with a path for transitioning MSW waste to energy in cities and during emergencies.

## 1 Introduction

The coronavirus disease 2019 (COVID-19) pandemic exposed several shortcomings and constraints across global socio-economic, healthcare, and environmental systems (Nzediegwu and Chang, 2020). Millions of people suffering from this pandemic face serious health problems, and it has been linked to approximately 6.9 million deaths globally (Yousefi et al., 2021). Concurrently, the pandemic has brought short-term environmental benefits, including decreased air pollution, reduced noise, and enhancements in biodiversity and tourism sites.- Poorly managed waste can become a breeding ground for pathogens, increasing the risk of disease spread and environmental pollution, posing significant risks, as witnessed during the COVID-19 outbreak. Improper disposal and handling of escalating waste raised public concerns about potential secondary transmission routes and highlighted broader vulnerabilities in waste management systems (Sarkodie and Owusu, 2021). Practices such as open burning or uncontrolled incineration degrade air quality and pose severe health risks due to exposure to hazardous materials. Approximately 85%–90% of waste sent to open landfills typically causes severe environmental and health problems (Ouda et al., 2016). The problem of waste mismanagement underscores an urgent need to transition from landfilling to advanced waste-to-energy (WTE) technologies for the production of value-added products.

The pandemic highlighted the importance of sustainable waste management practices, particularly in light of the increased medical waste resulting from improper handling of municipal solid waste (MSW). Saudi Arabia currently produces 15 million tons of solid waste annually (Radwan et al., 2021). Projected waste generation in the Kingdom is expected to rise significantly, as Vision 2030 aims to increase the annual number of pilgrims from 8 million to 30 million (Nizami et al., 2017). This massive waste problem will hinder the Kingdom’s waste management, potentially posing risks of disease spread. WTE technologies offer a more sustainable solution, reducing landfill waste, generating renewable energy, and minimizing environmental impacts (Alao et al., 2022). The pursuit of energy recovery from waste streams is part of a broader effort to valorize industrial by-product streams, such as flue gas (Rowshanaie et al., 2022; Rowshanaie et al., 2015; Rowshanaie et al., 2020).

Gasification is a promising WTE method, converting MSW into syngas (a valuable energy carrier) usable for electricity production. Gasification is a thermochemical conversion method that transforms organic-rich waste into a combustible gas mixture, commonly known as syngas, under controlled oxygen and temperature conditions (Lee, 2022). The syngas primarily contains hydrogen (H_2_), carbon monoxide (CO), and methane (CH_4_), which can be further utilized for electricity or fuel production (Javed et al., 2025a). WTE technologies have the potential to reduce environmental and health impacts associated with traditional waste disposal, advancing sustainable development and the circular economy. However, this requires substantial investment in state-of-the-art technologies and infrastructure (Alao et al., 2022). Government support through funding and favorable policies can stimulate these initiatives, generate green jobs, and save millions of dollars annually in Saudi Arabia. Although the results of this study provide an overall techno-economic and environmental analysis of MSW gasification using a simulation-based modeling approach, this work has limitations and makes certain assumptions. The system boundary of this study is limited to a gate-to-gate approach and is based on secondary data sources. As a result, upstream collection and downstream distribution are not included. While this system boundary was assumed for scalability and policy relevance, it is also important to note that the results are a modeled projection and not actual operating plant data; performance must be further validated at pilot scale.

Innovative thermal treatments, such as catalytic gasification and plasma-assisted gasification, effectively convert MSW into high-quality syngas. Catalytic gasification enhances reaction rates, whereas plasma-assisted gasification uses high-temperature plasma for efficient syngas production (Bolívar Caballero et al., 2022; Ruiz-Martín et al., 2025). These methods offer improved efficiency, reduce tar formation, and increase syngas yield, representing promising solutions for sustainable energy production. Diverting waste from landfills to gasification mitigates environmental and health risks by providing cleaner alternatives to conventional disposal, preventing toxic emissions (Alaghemandi, 2024). Syngas primarily comprises hydrogen (H_2_) and carbon monoxide (CO), serving as precursors for the production of synthetic fuels and electricity (Rey et al., 2024). Integrating gasification into existing waste management frameworks can reduce environmental pollution and enhance energy security (Javed et al., 2025a). It can also provide critical energy during pandemics, offsetting increased energy demands in hospitals and quarantine facilities.

The feasibility and sustainability of syngas production are determined through a Life Cycle Assessment (LCA), an extensive assessment method based on the ISO 14040 and ISO 14044 standards. The economic feasibility and environmental concerns are assessed during the production process by the LCA (Collotta et al., 2019). It evaluates both the positive and negative impacts, as well as the technology implications and policy regulations (Pandit et al., 2025). Additionally, gasification aligns with the United Nations Sustainable Development Goals (SDGs), specifically SDG 3 (Good Health and Wellbeing), SDG 7 (Affordable and Clean Energy), SDG 12 (Responsible Consumption and Production), and SDG 13 (Climate Action). This study applies simulation-based modeling without experimental implementation. All data on gasifier performance, process parameters, and energy conversions are derived from established literature and credible databases (Javed et al., 2025a; Ouedraogo et al., 2021).

This research employs simulation-based LCA and techno-economic models, utilizing GaBi software, to comprehensively estimate environmental and economic indicators. The model evaluates syngas production under realistic, simulated conditions, avoiding the need for physical pilot plants. A case study in Jeddah, Saudi Arabia, illustrates how diverting MSW from landfills to a gasification facility provides a sustainable alternative. The study addresses three key objectives: (1) developing an optimized MSW-to-syngas production model, (2) conducting a detailed techno-economic feasibility assessment, and (3) extensively evaluating environmental impacts, including hotspot identification and sensitivity analysis. The novelty of this work lies in its integrated approach to assessing the viability of MSW gasification as a strategy for urban resilience. It uniquely incorporates a comprehensive set of sustainability indicators, including endpoint damage categories, normalized impacts, uncertainty analysis, full life-cycle costing, and assessment of by-product valorization. This approach not only provides energy resilience but also addresses a key public health vulnerability. Gasification of hazardous pandemic waste can significantly decrease the risk of secondary disease transmission by preventing its accumulation. This holistic framework demonstrates the potential of gasification to reduce landfill dependence and lower greenhouse gas emissions. It also positions as a robust technology for enhancing urban energy security and waste management resilience against public health crises and other systemic disruptions.

## 2 Materials and methods

### 2.1 Existing waste management system in Jeddah city: a case study

Jeddah, the second-largest city in the Kingdom, serves as a significant commercial hub in the Middle East. However, the city is struggling with a severe solid waste issue because of its rapidly growing population, which has reached 3.5 million (Waste management in jeddah EcoMENA, 2024). The municipal authority in Jeddah requires assistance in handling the mounting urban waste problem. Every day, over 5,000 tonnes (approximately 14% of total waste in Saudi Arabia) of solid waste are generated, making it imperative to address the issue promptly (Hegazy et al., 2024). In Jeddah, the process of solid waste management begins with the collection of waste from bins located in residential and commercial areas. The collected waste is then transported to a transfer station, where it is disposed of. Most MSW, with an estimated 1.5 million tons of waste annually, is disposed of in Buraiman’s landfill. The landfill is expected to last about 20–30 years (Alaghemandi, 2024). Reduction, reuse, recycling, and energy recovery are getting attention but are still in their early stages.

The informal sector plays a significant role in waste management and recycling, with a recycling rate of 10%–15%, which is largely attributed to the informal sector (Hidalgo-Crespo et al., 2023). Nearly 40% (including PPE, plastics, paper, cardboard, and wood) of the total waste in Jeddah can be gasified to produce syngas and energy (Hakami and Abu Seif, 2015). The informal sector collects recyclables, such as paper, metals, and plastics, from MSW. Currently, there are no large-scale recycling or energy recovery systems in operation in Jeddah. The lack of integrated waste management infrastructure, limited public awareness, and ineffective policy enforcement have hindered the transition to sustainable waste management (Zhang et al., 2024). This study utilizes waste composition and generation data from Jeddah City as a case study to simulate the environmental performance of a proposed gasification-based WTE model. All primary waste input values were modeled based on the city’s composition records, while process flows were developed using literature and GaBi datasets (Ruiz-Martín et al., 2025). Among various WTE technologies, gasification has shown strong potential due to its ability to handle heterogeneous MSW with lower environmental burdens compared to incineration (Gonçalves Mollica et al., 2024).

In this study, a simulation-based gasification model was selected as the core WTE approach, considering both technical feasibility and relevance to Saudi Arabia’s policy direction on a circular economy. The process includes sorting, shredding, gasification, and assumed energy recovery from syngas. These steps were not performed and constructed in GaBi software using a combination of localized waste input data and international inventory datasets (Ruiz-Martín et al., 2025). The system boundary was defined from the point of MSW reception at the facility to the output of usable syngas, excluding external collection and downstream electricity utilization. The decision to select gasification over incineration or anaerobic digestion was supported by its efficiency in volume reduction and its suitability for mixed waste streams with high moisture and organic content (Obileke et al., 2023).

### 2.2 Life cycle assessment (LCA)

This study followed the ISO 14040 and ISO 14044 standards to conduct LCA for the simulated syngas production system. LCA was applied as a structured decision-making tool to quantify environmental burdens for each process and to support an impact-based evaluation of the gasification route under simulated conditions. The four stages of LCA, namely goal and scope definition, inventory analysis, impact assessment, and interpretation, were applied to understand material flows, energy inputs, and the contribution of emissions to environmental indicators. The assessment included midpoint and endpoint impact categories, utilizing the ReCiPe methodology and simulation modeling with GaBi software. All data were normalized to a single functional unit, and the results were interpreted to identify major hotspots and system-level trade-offs. This LCA provided a more comprehensive picture of process sustainability and was a crucial step toward understanding the environmental performance of waste-to-syngas conversion in the face of pandemic challenges and disruptions.

### 2.3 Goal and scope

Goal and scope are the initial steps in any LCA study, as they clearly define the system boundary and limitations. The goal of the study was to assess the environmental and economic performance of syngas production from mixed MSW using a simulation-based LCA model. The broader objective was to examine how WTE technologies, mainly gasification, can reduce the risk of pandemic spread. It also aimed to evaluate circular economy strategies, particularly in the context of traditional systems disrupted by the public health crisis.

**FIGURE 1:**
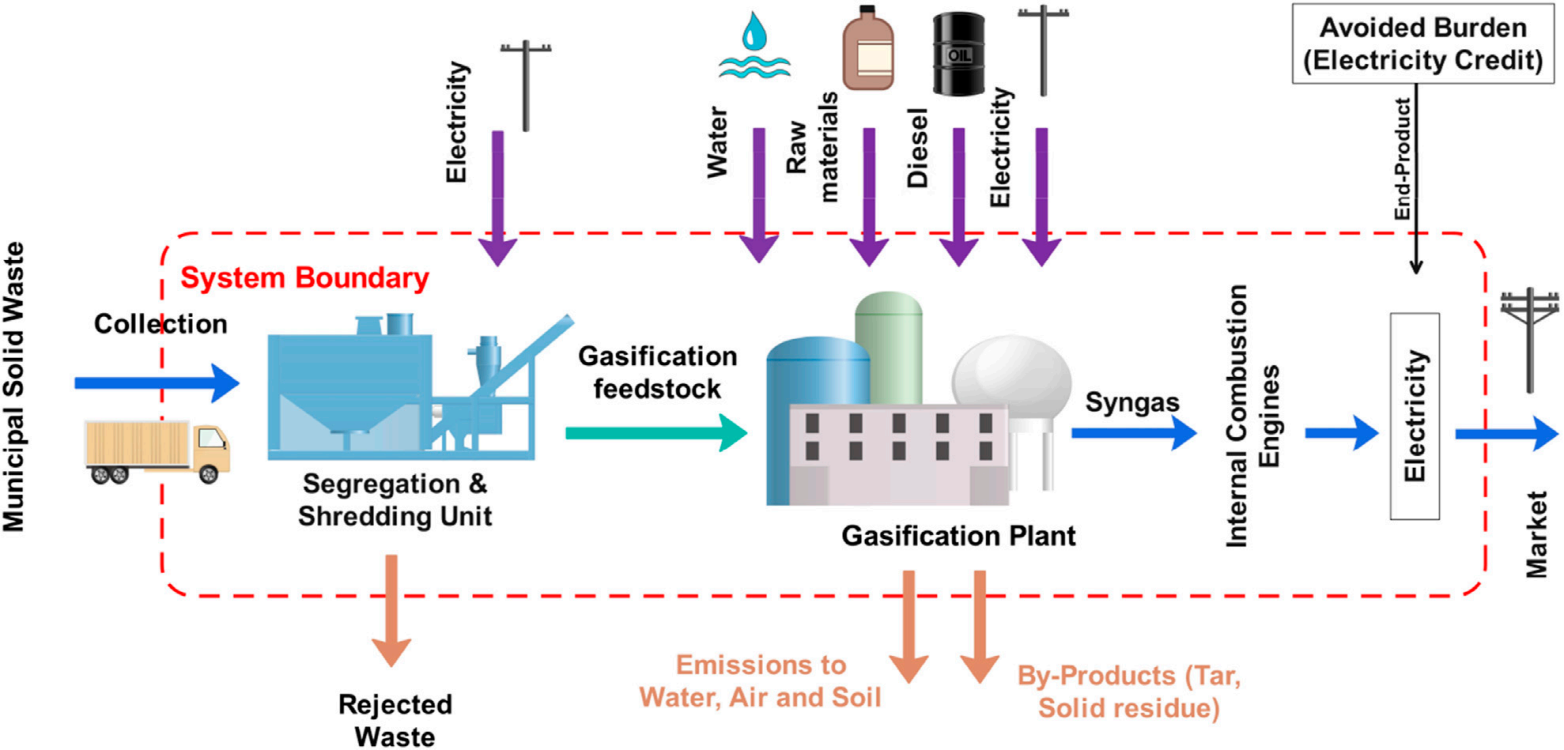
System Boundary of MSW gasification process.

A gate-to-gate boundary was adopted, focusing on all internal stages, including waste segregation, shredding, syngas production, and electricity generation through internal combustion engines (ICE). The system boundary, as illustrated in Figure 1, also accounts for all upstream and downstream flows related to electricity, diesel, water, and raw materials (e.g., air, acetone, propane, charcoal). Emissions to air, water, and soil, as well as by-products such as tar and solid residues, were included as output flows. The avoided burden approach was used for electricity credit, reflecting the environmental offset by displacing grid electricity.

The functional unit (FU) selected for the study is one metric ton of mixed MSW processed. All material and energy inputs, emissions, and outputs are normalized to this FU. The modeling assumptions are based on a 50 tons per day (TPD) gasification plant processing 18,000 tons of MSW annually. Primary waste composition data were collected from the Jeddah municipality, while technical inputs were sourced from the literature and life cycle inventory (LCI) datasets available in the GaBi software. For processing electricity inputs, the Electricity Grid Mix (EGM) dataset from GaBi was selected. This dataset, representing the Gulf Cooperation Council region, was chosen as the most appropriate proxy for Saudi Arabia’s grid. This choice was necessitated by the absence of a country-specific Saudi Arabian grid mix dataset within the GaBi software, and the GCC dataset was deemed the closest representation due to the shared energy infrastructure and predominant reliance on fossil fuels within the region. The ReCiPe 2016 (H) method was used for midpoint and endpoint impact assessment. This scope provided a comprehensive view of the energy recovery potential, environmental trade-offs, and cost structures associated with syngas production.

### 2.4 Inventory analysis and process description

In this study, the modeled gasification system was based on a total input of 1 ton of mixed MSW collected at the treatment gate in Jeddah, Saudi Arabia. The process was broken down into three primary stages to better represent the contribution of each activity to environmental loads. Primary data were derived from experimental and simulation studies of syngas production, mainly from Ouedraogo et al. (Ouedraogo et al., 2021). First, in the sorting and shredding phase, around 43.11 kWh of electricity was used to operate equipment for separating valuable materials and organic fractions. This stage resulted in approximately 400 kg of prepared feedstock being directed to the gasification process. This stage also separated 110 kg of mixed recyclables, including metals and glass, and approximately 490 kg of organic residue. In the next phase, gasification was modeled using fluidized bed parameters. The process involved approximately 0.61 kg of charcoal as a bed stabilizer, 1.51 L of propane (primarily for startup combustion), and 12.03 kWh of electricity for system operation. Additional inputs included 7.12 L of water and 1.78 L of acetone. Acetone is included on the basis of referenced LCI (Ruiz-Martín et al., 2025) and is presumed to account for solvent use in periodic equipment cleaning or maintenance operations. The air used for gasification was calculated to be approximately 10.4 Nm^3^ per ton of waste. The resulting product flow yielded approximately 564 Nm^3^ of syngas, along with 17.36 kg of tar and 30.82 kg of solid residue.

Finally, in the modeled recovery phase, syngas was assumed to generate 274.3 kWh of electricity. All values were based on secondary sources, the GaBi database, and regionally applicable modeling assumptions (Nzediegwu and Chang, 2020; Javed et al., 2025a; Ouedraogo et al., 2021). One of the critical challenges while performing LCA is ensuring that the data collected, whether from the field or published literature, is accurate and reliable enough (Olanrewaju et al., 2024). Waste composition data were taken from local records, while operational assumptions were mainly derived from published studies and GaBi process datasets. The system was modeled as a network of interlinked flows, where each input and output is part of a larger material balance. Table 1 provides a comprehensive overview of all inputs that entered the system from the environment, as well as the main outputs that were released or recovered during every step of the process. This structure ensures that the simulation remains consistent, balanced, and robust enough for a detailed environmental evaluation.

**TABLE 1:** Inventory table of gasification process (Ouedraogo et al., 2021).

Input/Output	Material	Unit	Value
MSW sorting and shredding			
Inputs	MSW Waste	ton	1
	Electricity	kWh	43.11
Outputs	Gasification feedstock	kg	400
	Metal, Glass, and unspecified waste	kg	110
	Organic waste	kg	490
Gasification			
Inputs	Gasification feedstock	kg	400
	Charcoal	kg	0.61
	Propane	L	1.51
	Electricity	kWh	12.03
	Water	L	7.12
	Acetone	L	1.78
	Air	Nm^3^	104.02
Outputs	Syngas	Nm^3^	564
	Tar	kg	17.36
	Solid Residue	kg	30.82
End-Product	Electricity	kWh	274.3

### 2.5 Life cycle impact assessment (LCIA)

A life cycle impact assessment was conducted to evaluate the environmental impacts associated with syngas production and electricity generation using GaBi version 10.0.0.71. The ReCiPe Midpoint (H) 2016 method was selected for environmental impact assessment. This is a reliable standard for environmental impact assessment due to its wide acceptance and comprehensive coverage of impact categories (Raluy and Dias, 2021; Life cycle assessment, 2025; Goedkoop and Heijungs, 2025). This method categorizes environmental impacts into 18 distinct categories, each linked to specific impacts (Huijbregts et al., 2017; Dong and Ng, 2014). Only twelve impact categories were selected based on the relevance of syngas production and electricity generation from MSW gasification.

Selected categories are climate change as global warming potential (GWP), Fine particulate matter formation potential (FPMFP), fossil depletion potential (FDP), freshwater consumption potential (FWCP), freshwater eutrophication potential (FWEP), human toxicity potential, cancer (HTP^c^), ionizing radiation potential (IRP), marine ecotoxicity potential (METP), metal depletion potential (MDP), photochemical ozone formation potential, ecosystem (POFP^eco^), stratospheric ozone depletion potential (SODP), and terrestrial acidification potential (TAP).

Midpoint and endpoint assessments, hotspot identification, and sensitivity analysis were used to thoroughly examine the environmental impacts of the study. All environmental effects were evaluated through a systematic process of data processing, including characterization of midpoint impacts, normalization of midpoint impacts, and weighting methods. Normalization and weighting enabled comparative analysis of all midpoint and endpoint impacts. The total impacts were further divided into three main damage categories, including human health, ecosystem degradation, and resource depletion.

Endpoint assessment encompasses the Disability-Adjusted Life Year (DALY) for human health, species loss per year due to ecosystem degradation, and the economic value associated with resource depletion (Kawahara et al., 2025). This organized method provides a comprehensive understanding of the environmental burdens associated with each area of protection, ensuring the effectiveness and detail of the environmental impact analysis. However, the systematic model was intended to examine processes and classify key contributors to environmental impacts. This model assessed the most impactful phases and dominant contributions through hotspot analysis and other distributive methods. Sensitivity analysis was also executed by varying material percentages by ±10% to ensure the model’s trustworthiness and practicality.

### 2.6 Interpretation

The interpretation phase is the final stage of the LCA process, where all results, assumptions, and uncertainties are consolidated to assess the overall performance of the system. In this study, different analyses were employed to achieve a reliable interpretation of the syngas production system. Midpoint results were calculated to evaluate the process-specific categories, such as GWP, FPMFP, FDP, FWCP, FWEP, HTP^c^, IRP, METP, MDP, POFP^eco^, SODP, and TAP. These outcomes helped to identify where the process was environmentally beneficial and where improvements were needed. The endpoint results were then used to summarize damage in broader areas, such as human health, ecosystem quality, and resource depletion. Endpoint assessment helped to understand the long-term impacts of the system.

Normalization analysis was used to assess the relative strength of each category compared to the others. Contribution analysis was also conducted, utilizing four major inputs (electricity, air, acetone, and propane) to determine which one was most responsible for environmental impacts. Electricity was the highest contributor in most categories. Uncertainty analyses, including sensitivity analysis and Monte Carlo analysis, were employed. Sensitivity analysis was conducted to examine a ±10% variation in each key input that affected the results. A Monte Carlo simulation was performed to verify the robustness of the midpoint results and the variation range. These comprehensive analyses enhance understanding of the modeled system and confirm that the results are both consistent and meaningful (Loucks et al., 2017).

### 2.7 Techno-economic assessment

Economic modeling was conducted to assess the economic viability of producing syngas using a combined approach of life cycle costing (LCC) and economic performance indicators. The LCC included the total internal costs (IC) and external costs (EC) (Zhou et al., 2021; Ahmad et al., 2025). Hence, the IC includes both initial capital investment (ICI) and operational costs. The ICI cost comprises the purchasing and construction costs. Moreover, operational costs included depreciation costs (C_d_), raw material costs (C_r_), utility costs (C_u_), management costs (CM), labor costs (C_l_), and maintenance costs (C_m_) (Lv et al., 2019; Musharavati et al., 2025). The IC was projected using the following Equation 1.

$$\text{IC}=C_{d}+C_{r}+C_{u}+\text{CM}+C_{l}+C_{m}$$ (1)

Therefore, during various phases of the life cycle process, the external cost signifies direct and indirect emissions into the environment. These emissions were calculated by multiplying the emission rate by a specific coefficient derived by Pa et al. (2013), as defined in Equation 2.

$$\text{EC}=\sum_{k=1}^{7}Ck\times Ek,lc$$ (2)

Ck remains a coefficient, as shown in the literature (Pa et al., 2013; Javed et al., 2025b). Ek, lc symbolizes the emissions linked with various life cycle stages. These emission values were obtained from the GaBi software database [36, . LCC is then calculated by summing the internal and external costs by using Equation 3.

$$\text{LCC}=\text{Internal cost }\left(\text{IC}\right)+\text{External cost }\left(\text{EC}\right)$$ (3)

Moreover, economic indicators, including net present value (NPV), net annual revenue (NAR), payback period (PB), and return on investment (ROI), were used to assess the project’s economic viability. Equations 4–7 calculate these economic indicators.

$$\text{NAR}=\left(\text{Annual Sales}\right)-\left(\text{Annual Investment}\right)$$ (4)

$$\text{NPV}=\frac{Rt}{\left(1+i\right)t}$$ (5)

$$\text{ROI}=\frac{Sum\;of\;all\;Profits*100\%}{Initial\;Investment}$$ (6)

$$\text{PB}=\text{last negative yr}+\frac{\text{CCF}\;\text{of}\;\text{last}\;\text{negative}\;\text{yr}}{\text{CCF}\;\text{of}\;\text{next}\;\text{year}}$$ (7)

Rt signifies the remaining cash flow at a given time t, i represents the reduction rate, and t represents the time of the cash flow.

## 3 Results

### 3.1 Midpoint impact assessment results

Midpoint results were calculated using the ReCiPe 2016 method, as shown in Table 2, with a focus on selected categories that are more relevant to gasification systems. The GWP of the overall modeled process was estimated at −100 kg CO_2_ eq., indicating a net climate benefit due to the electricity credit outweighing the process emissions. Similarly, the FDP showed a net result of −3.25E+01 kg of oil equivalent, indicating that the system avoided fossil resource use by offsetting energy requirements. FPMFP and TAP were also negative overall, reported at −2.50E–02 kg PM2.5 eq. and −8.49E–02 kg SO_2_ eq. respectively, primarily due to the substitution of electricity from syngas recovery.

**TABLE 2:** Midpoint results of environmental impact categories.

Categories	Abbreviations	Unit	Gasification process	Electricity credit	Net results
Climate Change	GWP	kg CO_2_ eq	3.67E+01	−1.37E+02	−1.00E+02
Fine particulate matter formation	FPMFP	kg PM2.5 eq	1.12E-02	−3.62E-02	−2.50E-02
Fossil depletion	FDP	kg oil eq	1.49E+01	−4.73E+01	−3.25E+01
Freshwater consumption	FWCP	m3	8.90E-02	−1.05E-01	−1.60E-02
Freshwater Eutrophication	FWEP	Kg P eq	2.08E-05	−2.12E-06	1.87E-05
Human toxicity, cancer	HTPc	kg 1,4- DB eq	8.59E-03	−2.27E-02	−1.41E-02
Ionizing radiation	IRP	kBq Co-60 eq. to air	4.24E-01	−3.68E-03	4.20E-01
Marine ecotoxicity	METP	kg 1,4- DB eq	8.65E-03	−1.67E-02	−8.05E-03
Metal depletion	MDP	kg Cu eq	1.48E-02	−2.50E-02	−1.02E-02
Photochemical ozone formation, ecosystem	POFPeco	kg NOx eq	2.19E-02	−5.59E-02	−3.40E-02
Stratospheric ozone depletion	SODP	kg CFC-11 eq	3.45E-06	−4.20E-06	−7.48E-07
Terrestrial acidification	TAP	kg SO_2_ eq	3.71E-02	−1.22E-01	−8.49E-02

METP and FWEP reported marginal net values −8.05E–03 and 1.87E–05 kg 1,4-DB eq. reflecting minor upstream chemical inputs, such as acetone and propane. For HTPc, the modeled system reported a net emission of −1.41E–02 kg 1,4-DB eq., again driven by upstream electricity savings. IRP, on the other hand, remained positive at 4.20E–01 kBq Co-60 eq. to air, likely because of background emissions associated with grid-based energy generation. The MDP and SODP both showed slight environmental benefits, recorded at −1.02E–02 kg Cu eq. and −7.48E–07 kg CFC-11 eq. respectively. POFPeco was calculated as −3.40E–02 kg NO_x_ eq., attributed to avoided combustion emissions. Water consumption, as reflected by FWCP, was moderately low, with a net result of −1.60E–02 m^3^, suggesting that the modeled system recovers more than it uses at the midpoint scale.

### 3.2 Normalized midpoint assessment

**FIGURE 2:**
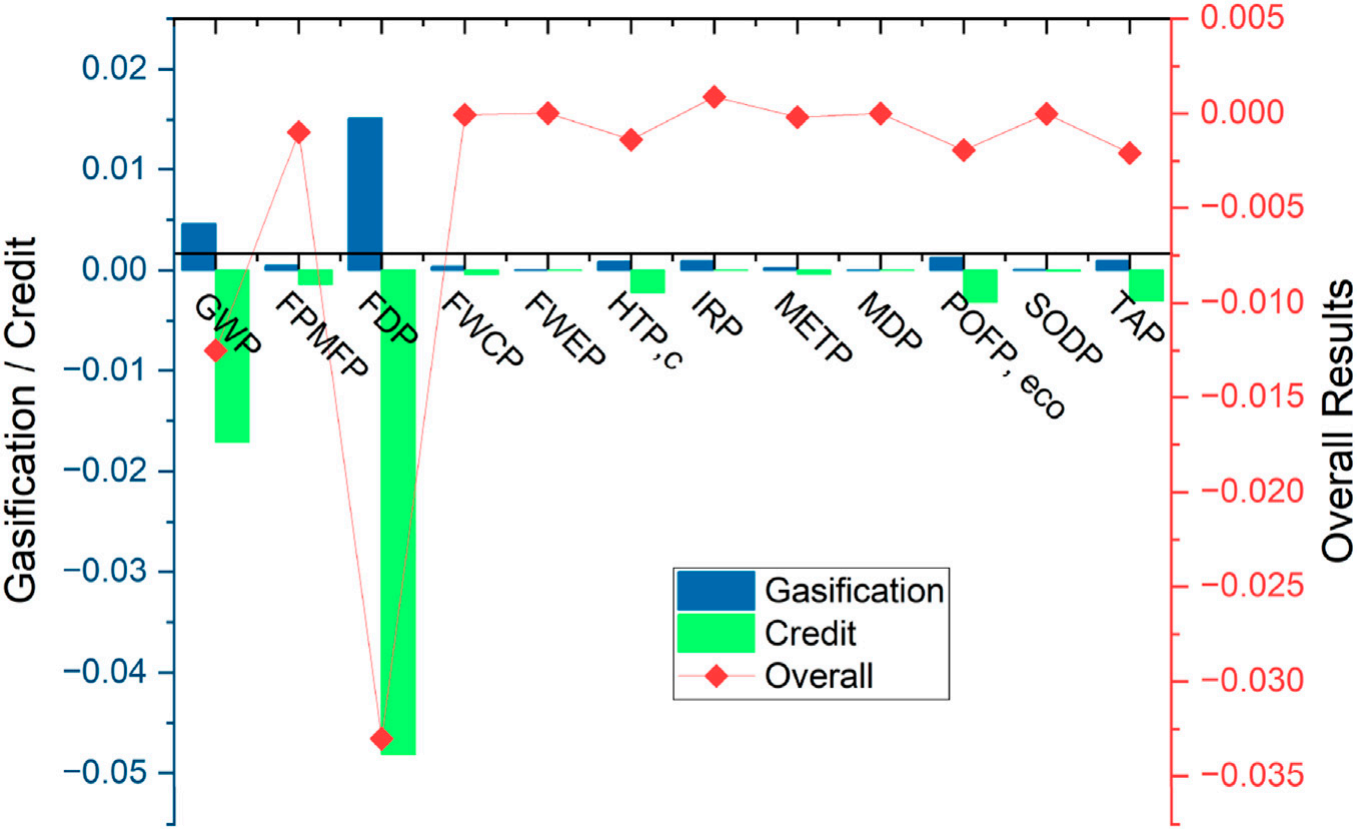
Normalization results of MSW gasification.

Normalization was applied to the selected midpoint categories to enable a clearer comparison of environmental impacts across different units and scales. The normalized results (as shown in Figure 2) highlighted the relative magnitude of each impact category after normalization. The process separates contributions from gasification, electricity credit, and overall net impact. It can be seen that most of the environmental load is offset through energy credit, resulting in negative overall values for several indicators. Among all the categories, FDP stood out as the most dominant contributor, with a normalized gasification value of around 0.0351 and an overall net score of −0.033. This is primarily due to the embedded energy in startup fuels, such as propane, and the use of upstream electricity in pretreatment. The GWP also made a significant contribution, with a normalized gasification value of 0.0046 and an overall net value of −0.0125. This showed that the emission reductions from electricity recovery outweigh the emissions during the gasification process.

FPMFP, POFPeco, and TAP also showed moderate normalized impacts from gasification, but with electricity credits reversing them into net environmental benefits. For example, POFP had a normalized process value of 0.0012 and an overall result of −0.00192. IRP and HTPc both contributed minor values from the gasifier but were mostly offset by avoided upstream burdens. Freshwater-related categories, such as FWCP and FWEP, showed minimal contributions, with net normalized values close to zero (−0.000062 and −0.000028, respectively), indicating that the modeled system consumes and offsets similar amounts of water-based emissions.

MDP and METP remained among the least impactful categories, which is expected since the system does not rely on heavy metals or high-toxicity chemicals. The normalization process facilitated the identification of the most sensitive categories affected by syngas production, confirming that gasification combined with electricity recovery yields net benefits in most of the selected environmental indicators.

### 3.3 Endpoint impact assessment

The endpoint indicators were calculated to assess the broader damage impacts across human health, ecosystems, and resource availability using the ReCiPe 2016 method. As shown in Table 3, the results represented a combination of direct gasification process burdens and environmental benefits resulting from electricity substitution. For human health, the total net impact was found to be −1.09E–04 DALY, indicating an overall benefit. Climate change was the leading contributor here, with 3.40E–05 DALY from gasification offset by −1.27E–04 DALY in credit, giving a net benefit of −9.30E–05 DALY. Other relevant categories, like fine particulate matter formation and photochemical ozone formation, showed smaller net health effects of −1.58E–05 and –2.96E–08 DALY, respectively. Human toxicity showed a marginal net increase (3.65E–08 DALY), while freshwater consumption and ionizing radiation also reflected small positive balances.

**TABLE 3:** Endpoint results of MSW gasification.

Categories	Units	Gasification process	Electricity credit	Net results
Overall Impacts (Human Health)	DALY	4.12E-05	−1.50E-04	−1.09E-04
Climate change	DALY	3.40E-05	−1.27E-04	−9.30E-05
Fine Particulate Matter Formation	DALY	7.05E-06	−2.28E-05	−1.58E-05
Freshwater Consumption	DALY	5.91E-08	−3.31E-09	5.58E-08
Human toxicity	DALY	1.41E-07	−1.04E-07	3.65E-08
Ionizing Radiation	DALY	3.61E-09	−3.12E-11	3.57E-09
Photochemical Ozone Formation	DALY	1.93E-08	−4.89E-08	−2.96E-08
Stratospheric Ozone Depletion	DALY	1.83E-09	−2.23E-09	−3.98E-10
Overall Impacts (Ecosystem)	species.yr	1.17E-07	−4.17E-07	−3.00E-07
Climate change	species.yr	1.03E-07	−3.83E-07	−2.81E-07
Freshwater Consumption	species.yr	2.31E-10	−1.01E-10	1.31E-10
Freshwater ecotoxicity	species.yr	1.11E-12	−1.03E-12	8.39E-14
Freshwater Eutrophication	species.yr	1.40E-11	−1.42E-12	1.26E-11
Land use	species.yr	3.57E-09	−3.49E-10	3.22E-09
Marine ecotoxicity	species.yr	9.10E-13	−1.75E-12	−8.40E-13
Marine Eutrophication	species.yr	3.02E-13	−5.43E-14	2.47E-13
Photochemical Ozone Formation	species.yr	2.83E-09	−7.21E-09	−4.39E-09
Terrestrial Acidification	species.yr	7.86E-09	−2.59E-08	−1.80E-08
Terrestrial ecotoxicity	species.yr	3.05E-11	−2.89E-12	2.75E-11
Overall Impacts (Resources)	USD	4.21E+00	−1.43E+01	−1.01E+01
Fossil depletion	USD	4.21E+00	−1.43E+01	−1.01E+01
Metal depletion	USD	3.85E-03	−6.09E-03	−2.24E-03

In the ecosystem damage category, the total net impact was calculated as −3.00E–07 species·yr, driven mainly by avoided burdens in categories such as land use and marine ecotoxicity. Land use contributed 3.22E–09 species·yr net loss, while freshwater ecotoxicity remained minimal (8.39E–14 species·yr). The largest credit came again from climate-related categories, particularly climate change (−2.81E–07 species·yr) and terrestrial acidification (−4.39E–09 species·yr). Overall, the ecosystem effect of the modeled gasification system was favorable in most categories. Regarding resource damage, the endpoint result indicated a net monetary impact of −1.01E+01 USD, suggesting that the system generates more savings than losses when resource depletion is taken into account. This was primarily due to avoided fossil fuel consumption (–$ 1.01E+01 USD) and, to a lesser extent, reduced metal depletion (–$ 2.24E–03 USD). This means the modeled gasification route for MSW has not only avoided emissions but also delivered quantifiable resource conservation in economic terms.

**FIGURE 3:**
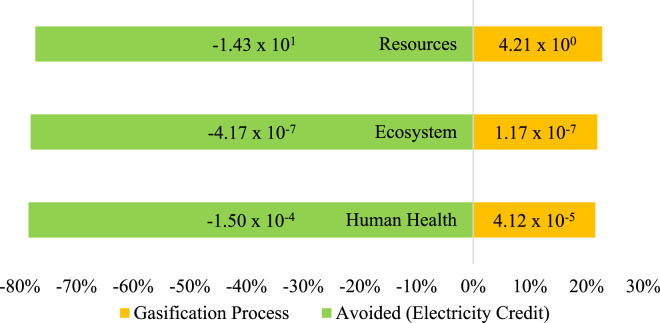
Endpoint Single score of MSW gasification.

When all three areas—human health, ecosystem quality, and resource depletion—are evaluated together, the net endpoint scores suggest that the process, which carries minor operational burdens, offers clear environmental and economic benefits. Figure 3 shows the single score of total endpoint results from the gasification process and the avoided burden.

### 3.4 Contribution analysis

**FIGURE 4:**
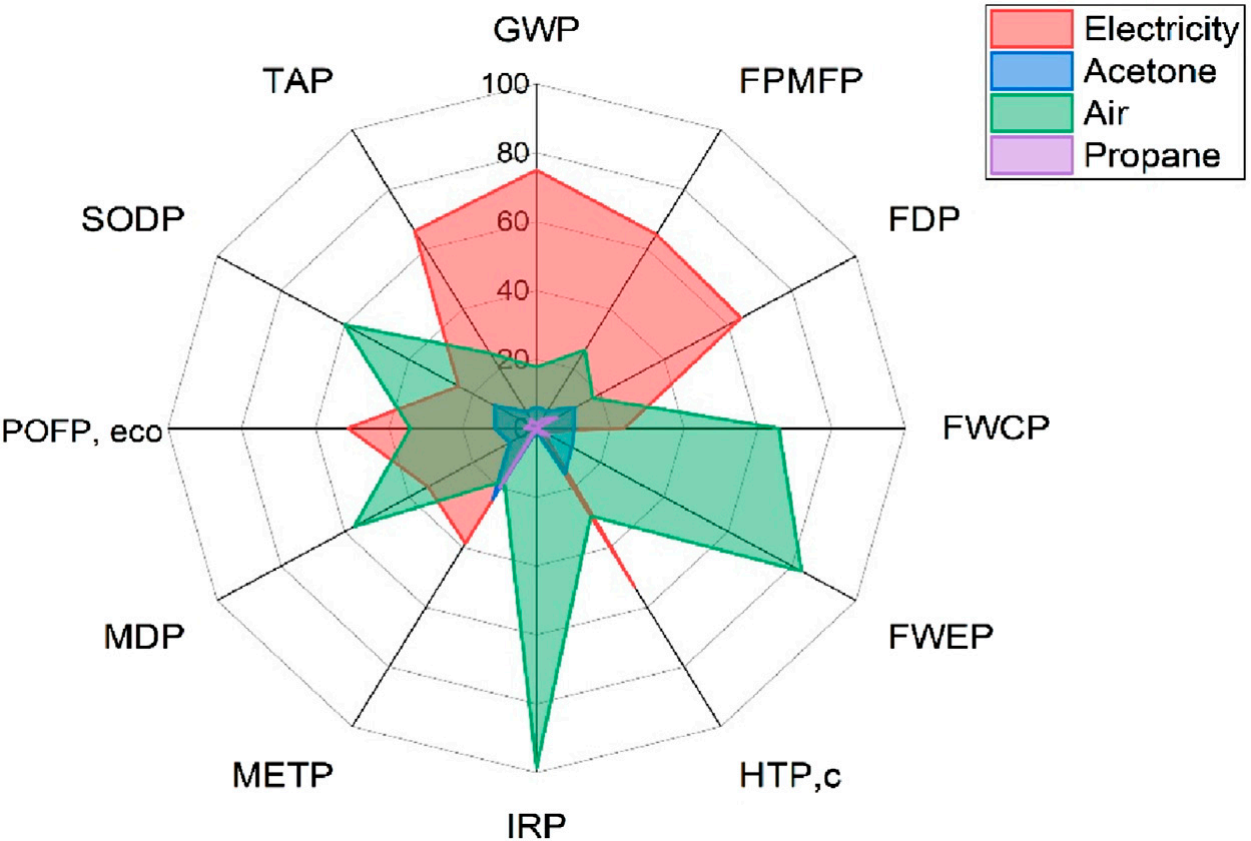
Contribution Analysis of MSW gasification process.

A contribution analysis was performed to identify which input flows contributed the most. The results are presented in Figure 4, which shows the percentage contributions from four key foreground inputs: electricity, acetone, air, and propane. The analysis showed that electricity was the single largest contributor in nearly all impact categories. For example, it accounted for 75% of GWP, 65% of FPMFP, and 64% of FDP. This trend highlights the significance of electricity consumption in the gasification and sorting phases, particularly in relation to its upstream emissions. Similarly, air input had a considerable share in categories such as FWEP (83.1%) and FWCP (65.7%). This suggests that modeled air flows, although technically emission-free, trigger background impacts through energy-associated datasets in GaBi.

Propane and acetone played more specific roles. Propane had higher contributions in categories like FDP (6.3%) and METP (19.9%), likely due to its fossil-based origin and toxic emissions during startup. Acetone showed peak impacts in IRP (98.6%) and POFP (11.4%), reflecting the upstream processing burden of solvent production. In HTPc and METP, acetone and propane together contributed over 35% of the impact. Interestingly, TAP and POFPeco were relatively more balanced across inputs, with electricity remaining the dominant contributor; however, other inputs, such as air and solvents, had noticeable shares.

The SODP and IRP categories were mainly influenced by acetone use, while FWEP and FWCP were shaped by high air input and moderate electricity demand. Overall, this contribution breakdown confirmed that electricity remains a dominant input in thermochemical modeling studies (even when syngas is assumed to recover power later). Upstream burdens from chemicals such as propane and acetone should not be underestimated. Several categories, such as IRP and METP, might be significantly reduced if cleaner solvents or low-carbon fuels were used instead.

#### 3.4.1 Comparative contribution of electricity and operating materials

**FIGURE 5:**
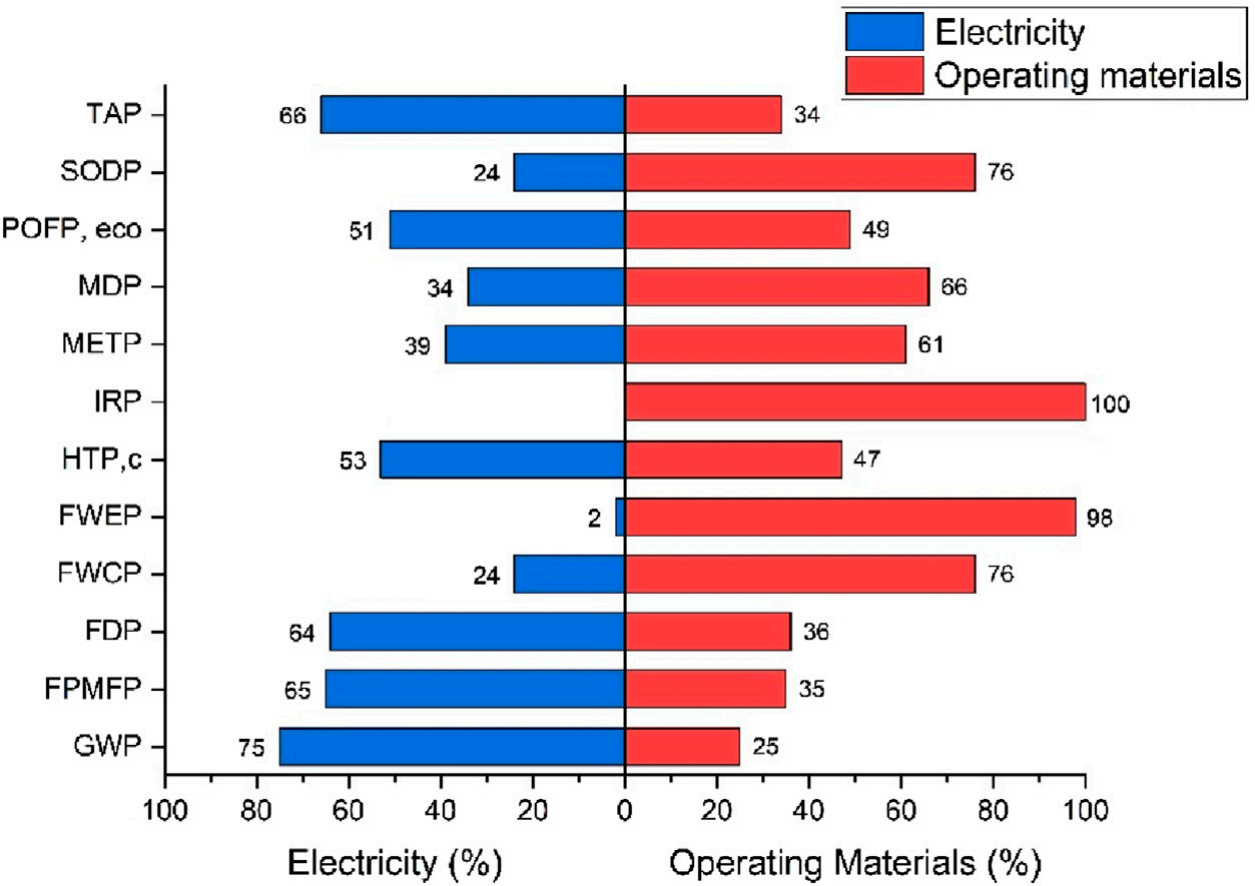
Comparative contribution of electricity and operating materials.

In addition to the detailed contribution analysis presented in Section 3.4, a comparative evaluation was conducted to quantify the relative contributions of electricity and operating materials across the midpoint environmental impact categories. This analysis aimed to deepen the understanding of input-specific burdens, with a particular focus on identifying the dominant environmental drivers among foreground activities. Figure 5 presents a visual breakdown of the percentage share of electricity (shown in blue) versus all operating materials (shown in red) across 12 selected midpoint categories. Results reveal that electricity is the most significant contributor in several categories, including GWP (75%), FPMFP (65%), and FDP (64%). These outcomes underscore the environmental intensity of electricity use in the gasification and pre-processing stages, even when the electricity is partially offset through recovery from syngas.

On the other hand, operating materials, comprising water, acetone, propane, charcoal, and air, demonstrated higher contributions in categories such as IRP (100%), FWEP (98%), and SODP (76%). This highlights the environmental load embedded in the procurement and upstream production of these inputs, particularly solvents and air compression systems. The balance observed between electricity and materials in categories such as POFPeco (51% vs. 49%) and HTPc (53% vs. 47%) further supports the multifaceted nature of environmental impacts in WTE systems. Such insights are crucial for targeted optimization, indicating that reductions in both energy intensity and material input complexity are necessary to enhance overall sustainability. This dual-layered analysis enhances the robustness of the interpretation phase by showing not only that inputs are critical but also how their interplay affects environmental trade-offs.

### 3.5 Uncertainty analysis results

#### 3.5.1 Sensitivity analysis

A sensitivity analysis was conducted to examine the impact of a +10% in individual input flows, including acetone, air, electricity, and propane, on the midpoint environmental categories. The purpose was to determine which inputs were more responsible for shifting environmental performance, either positively or negatively. Table 4 presents the variation in percentage for each impact category. Among all inputs, electricity had the most consistent and highest effect on the overall impacts. A 10% change in electricity impacted FDP by 2.93%, FPMFP by 2.90%, TAP by 2.88%, and GWP by 2.74%. This is particularly evident for GWP, where a +10% change in electricity use leads to a +2.74% change in the net result. This strong positive correlation underscores the carbon footprint of gasification. Other categories, such as HTPc and POFPeco, also showed sensitivity at +3.24% and +3.30%, respectively. Electricity also had a significant impact on MDP, with a variation of +4.91%. These variations were mainly due to the high upstream burden associated with electricity use in sorting and thermal operations.

**TABLE 4:** Sensitivity analysis of key inputs of gasification process.

Inputs	Acetone	Air	Electricity	Propane
Variations	+10%	+10%	+10%	+10%
GWP	+0.23%	+0.65%	+2.74%	+0.04%
FPMFP	+0.26%	+1.17%	+2.90%	+0.13%
FDP	+0.55%	+0.81%	+2.93%	+0.29%
FWCP	+0.23%	+10.60%	+0.21%	+0.01%
FWEP	+1.22%	+9.25%	+0.23%	+0.43%
HTP,c	+0.94%	+1.79%	+3.24%	+0.13%
IRP	+0.10%	+9.95%	+0.02%	+0.02%
METP	+2.58%	+1.91%	+4.18%	+2.15%
MDP	+1.22%	+8.27%	+4.91%	+0.06%
POFP, eco	+0.74%	+2.21%	+3.30%	+0.20%
SODP	+6.09%	+27.70%	+11.25%	+0.96%
TAP	+0.25%	+1.10%	+2.88%	+0.13%

The air input had a significant impact, particularly in freshwater-related categories, with FWCP increasing by +10.60%, FWEP by +9.25%, and IRP by +9.95%. POFP also changed by +2.21%, and METP by +1.91%. These results suggest that air compression and circulation, as modeled through GaBi databases, have energy demands that result in indirect emissions through multiple impact pathways. Acetone exhibited moderate changes in categories such as METP (+2.58%), MDP (+1.22%), FWEP (+1.22%), and POFP (+0.74%). Notably, this led to the highest variation in SODP at +6.09%, likely due to the chemical precursors involved in solvent manufacturing. Although the absolute quantity of acetone in the process was low, its upstream chemical intensity still made a measurable impact.

Propane was the least sensitive input across all primary inputs. Most categories showed variations +0.30%, with GWP at just +0.04%, FPMFP at +0.13%, and FWCP at +0.01%. Only METP (+2.15%) and FWEP (+0.43%) were slightly affected due to the fossil-based nature of the fuel, which was only used during system startup. Overall, the results show that electricity and air are the two most influential inputs in this system. These findings support that reducing energy consumption or sourcing greener electricity could significantly improve the environmental performance of syngas production.

#### 3.5.2 Monte carlo analysis

A Monte Carlo simulation was performed to assess the uncertainty and variability across selected midpoint categories. The model employed probabilistic sampling to estimate the 10th and 90th percentile bounds and standard deviation (SD) for each impact result based on the system inputs and their associated background datasets. The basic scenario results are listed in Table 5, along with the spread ranges, which demonstrate the model’s robustness under small fluctuations. Most categories showed low to moderate uncertainty. For example, GWP had a basic value of −1.00E+02 kg CO_2_ eq., with a standard deviation of −2.55%. Its 10th and 90th percentile results were −1.03E+02 and −9.73E+01 kg CO_2_ eq., which shows narrow variability and a stable central trend. FPMFP and FDP also showed tight ranges with SDs of −2.85% and −2.81% respectively.

**TABLE 5:** Monte Carlo analysis of MSW gasification.

Categories	Unit	Basic scenario	SD	10%	90%
GWP	kg CO_2_ eq	−1.00E+02	−2.55%	−1.03E+02	−9.73E+01
FPMFP	kg PM2.5 eq	−2.50E-02	−2.85%	−2.59E-02	−2.43E-02
FDP	kg oil eq	−3.25E+01	−2.81%	−3.35E+01	−3.16E+01
FWCP	m3	−2.82E+01	−9.08%	−3.13E+01	−2.43E+01
FWEP	kg P eq	1.87E-05	7.99%	1.68E-05	2.06E-05
HTP,c	kg 1,4-DB eq	−1.41E-02	−3.52%	−1.47E-02	−1.35E-02
IRP	kBq Co-60 eq. to air	4.20E-01	8.51%	3.73E-01	4.69E-01
METP	kg 1,4-DB eq	−8.00E-03	−5.08%	−8.56E-03	−7.56E-03
MDP	kg Cu eq	−1.02E-02	−8.78%	−1.14E-02	−9.00E-03
POFP, eco	kg NO_x_ eq	−3.40E-02	−3.71%	−3.55E-02	−3.25E-02
SODP	kg CFC-11 eq	−7.49E-07	−27.30%	−1.01E-06	−4.56E-07
TAP	kg SO_2_ eq	−8.50E-02	−2.81%	−8.78E-02	−8.25E-02

Water-based indicators like FWCP showed slightly higher uncertainty, with an SD of −9.08% and a basic value of −2.82E+01 m^3^. The 10% and 90% values stretched from −3.13E+01 to −2.43E+01 m^3^, showing a wider range of spread, likely due to variability in electricity and air use upstream. IRP had the highest standard deviation, recorded at 8.51%, with the basic value of 4.20E–01 kBq Co-60 eq. to air. This level of uncertainty may come from fluctuations in background power grid emissions. SODP was the most unstable category, with a very large standard deviation (SD) of −27.30%. The basic result was −7.49E–07 kg CFC-11 eq., but its spread from −1.01E–06 (10%) to −4.56E–07 (90%) showed that any small change in acetone or related chemical flows could significantly shift the output. Other categories, such as HTPc (−3.52%), POFPeco (−3.71%), and TAP (−2.81%), remained within normal fluctuation bands, suggesting their results are relatively dependable.

Monte Carlo outcomes confirmed that the model is reasonably stable for most midpoint indicators, with only a few exceptions showing wide sensitivity. These results support the reliability of the LCA model under the modeled assumptions but also highlight the importance of selecting accurate background data, especially for impact categories driven by solvent use or electricity generation pathways.

### 3.6 Techno-economic assessment

The techno-economic analysis involved the LCC and economic parameters, as outlined in Equation 3 from the methodology section. The case study was conducted to assess the economic feasibility of a 50 TPD MSW gasification plant, considering typical operating conditions in Jeddah, Saudi Arabia. The analysis assumes the implementation of a medium-scale syngas production facility capable of processing 50 TPD of MSW, operating for 360 days annually. This capacity is aligned with the waste availability in urban centers such as Jeddah, which generates high volumes of organic and mixed waste fractions. According to the inventory (Table 1), processing 1 ton of MSW yields approximately 274.3 kWh of electricity. Over the course of a year, the plant would generate a total of 4,937,400 kWh of electricity, which, at a market sale price of $0.08 per kWh, would result in an annual electricity revenue of approximately $394,992.

Additionally, gasification of 18,000 tons of MSW produces valuable by-products, including approximately 312,480 kg of tar and 554,760 kg of solid residue. Assuming updated market prices of USD 0.30/kg for tar and USD 0.05/kg for solid residue, these by-products generate annual revenues of USD 93,744 and USD 27,738, respectively. Hence, the total annual revenue from the plant is estimated at USD 516,474. The Internal Capital Investment (ICI) required to establish the facility is estimated at USD 1 million, which includes USD 800,000 for equipment procurement and USD 200,000 for construction, as per quotations received from regional suppliers and local market rates. Depreciation of this capital over a 20-year plant lifespan with a 5% salvage value, calculated using the straight-line method, amounts to USD 47,500 per year.

Table 6 shows the internal cost assumptions for the TEA. Annual operating costs comprise expenditures for raw materials and utilities (USD 150,632), plant maintenance (USD 35,000), management (USD 30,000), and labor (USD 45,000), totaling USD 260,632. When depreciation is included, the total internal cost (IC) of the plant rises to USD 308,132 per year. Consequently, the net annual revenue (NAR) for the system is USD 208,342. Using a 10-year project life and an 8% discount rate, the Net Present Value (NPV) is calculated at USD 398,000. This value indicates that the plant would provide significant economic returns over its lifespan. Furthermore, the ROI over 10 years is estimated at 208.34%, reinforcing the project’s financial viability. The payback period is calculated to be approximately 4.8 years, which is reasonable for medium-scale energy recovery projects.

**TABLE 6:** Data for the calculation of economic analysis.

Component	Economic assumptions
Depreciation	Life period 20 years; Salvage value 5% (Zhou et al., 2017)
Equipment cost	USD 800,000 (Quotes by equipment supplier after customization)
Construction cost	USD 200,000 (Local Market rate)
Raw materials	Charcoal USD 0.49/kg, Propane USD 0.30/L, acetone USD 0.58/L, Water USD 0.013/L, Air USD 0.02/Nm3 (Local Market Rates)
Utilities cost	Electricity USD 0.08/kWh (Commercial rate), Water USD 0.013/L
Management Cost	USD 30,000 per annum (Local assumptions)
Labor cost	USD 45,000 per Annum (Local wages rate)
Maintenance cost	USD 35,000 per annum (Local assumptions)

**FIGURE 6:**
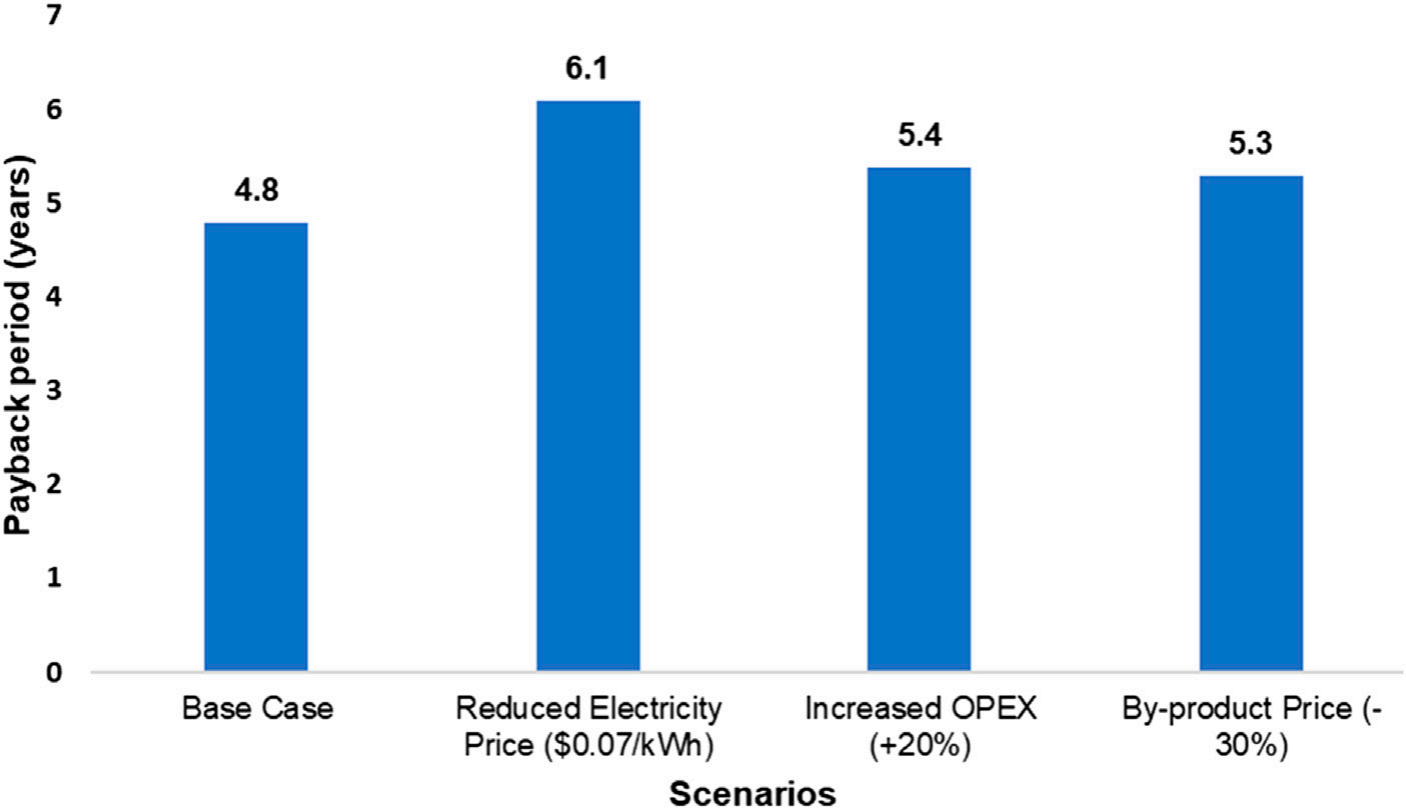
Payback period of different case scenarios.

A sensitivity analysis was also conducted to assess financial robustness under various market scenarios. If the electricity price dropped to USD 0.07/kWh, the payback period would extend to 6.1 years, and NPV would decrease to USD 150,000. In a scenario where operating and maintenance costs increase by 20%, the payback period would shift to 5.4 years, still within an acceptable range, as shown in Figure 6. In a scenario with a 30% decrease in the price of tar (to $0.21/kg) and solid residue (to $0.035/kg), the total annual revenue would decrease to $473,568. This would extend the payback period to 5.3 years and lower the NPV to approximately $350,000. A scenario assuming no market value for tar was analyzed to test an extreme boundary condition. This resulted in a payback period of 5.8 years and an NPV of $280,000. While this extends the payback, but the project remains financially viable, demonstrating a strong buffer against the complete failure of the tar market. Conversely, a 30% price increase would shorten the payback period to 4.4 years. This analysis confirms that the project’s economic viability is robust to fluctuations in by-product markets, maintaining a compelling financial case even under conservative assumptions.

Even with a 10% reduction in tax revenue, the plant would achieve payback in 5.2 years with an NPV of USD 340,000. These scenarios indicate strong economic resilience. These results suggest that gasification of MSW for syngas production and subsequent electricity generation represents a feasible energy recovery strategy for urban centers with consistent waste streams. The recovered energy and material values help offset operational costs and provide financial incentives to transition from landfilling to thermal treatment. Although the project incurs internal costs, its revenue-generating capacity, relatively short payback period, and high NPV make it an attractive solution for sustainable waste management in Saudi Arabia. If supported by favorable policy instruments, such as feed-in tariffs, renewable energy credits, or municipal subsidies, the scalability of such systems could transform waste management infrastructure.

Based on the external cost coefficients presented in Table 7, the net external cost saving associated with the gasification of MSW is calculated as −2.79 USD per ton of waste processed. When scaled to the assumed annual capacity of 18,000 tons, this results in a total external environmental cost saving of approximately USD 50,220 per year. These negative external costs indicate an overall environmental benefit due to reduced emissions of key pollutants, including CO_2_, CH_4_, NO_x_, and SO_2_, which are either fully or partially offset by the gasification system. Incorporating this value into the broader LCC framework reinforces the economic and environmental viability of the system, highlighting the dual benefits of improved financial performance and reduced societal damage from emissions.

**TABLE 7:** External cost analysis of MSW gasification.

Pollutants	Emissions^a^ (kg)	Coefficient^b^	External cost^c^ (USD)
CO_2_	−80.00	0.03	−2.24
CH_4_	−0.56	0.21	−0.12
NO_x_	−0.03	4.47	−0.13
CO	−0.02	0.59	−0.01
NMVOC	−0.02	1.24	−0.03
PM	0.00	10.13	0.00
SO_2_	−0.07	3.41	−0.25
		Total	USD -2.79/ton

a GaBi and b,c (Loucks et al., 2017).

## 4 Discussion

### 4.1 Comparative analysis with existing studies of the syngas production system

Various studies have been conducted on the gasification of different types of MSW, but a consistent approach that combines both environmental and economic aspects remains lacking. This study primarily focuses on mixed MSW and its conversion into syngas under pandemic-influenced conditions, which stands out compared to older literature in multiple ways. For instance, Miandad et al. (2017) and Javed et al. (2025a) investigated the generation of syngas from plastic waste, highlighting significant environmental concerns, including high greenhouse gas (GHG) emissions, acidification, and human toxicity. In contrast, this study utilized an updated life cycle inventory, and the ReCiPe method found relatively moderate emissions when mixed MSW is sorted and properly pretreated before gasification.

Several recent works, such as those by Radwan et al. (2021) and Miandad et al. (2016), have also explored the gasification process for energy recovery. However, most research focused on either technical optimization or LCA midpoint assessments. This study’s unique point is the integration of midpoint, endpoint, normalized impacts, and full-scale LCC. Additionally, some existing literature overlooked economic indicators such as net annual revenue (NAR), ROI, and external cost savings, key parameters that we assessed in detail. It demonstrates the practical application of implementing syngas-based WTE in cities like Jeddah.

Notably, earlier studies also did not consider pandemic-related waste behavior, which actually changed waste compositions and added new challenges. It is important to distinguish that this study modeled mixed MSW and not dedicated infectious or hazardous medical waste streams. The relevance for crisis preparedness lies in the demonstrated capability of gasification to safely process the heterogeneous mix of general solid waste, which, during a pandemic, can see a significant influx of challenging materials like plastics and contaminated packaging that may be improperly disposed of in the MSW stream. This capability helps prevent system failures and open dumping that exacerbate public health risks during emergencies, improving sanitation conditions.

Furthermore, older research overlooked the potential of tar and solid residue recovery, which could generate additional revenue streams. All in all, compared to prior works, this study provides a more realistic, pandemic-relevant, and economically viable outlook of syngas production through MSW gasification. The framework can serve as a reference for policy planning and for establishing decentralized energy units near medical or urban areas in times of crisis. Table 8 presents a comparative analysis of various studies on syngas production, each employing different assessment methods.

**TABLE 8:** Comparative analysis of different syngas production LCA studies.

Country	Feedstock	Assessment method/Software	System boundary	Functional unit	References
Denmark	MSW-organic	SimaPro 9	Gate-to-gate	1 kg of wet food waste	Kirkeby et al. (2006)
Italy	MSW	OpenLCA	Gate-to-gate	1 ton	Fruergaard and Astrup (2011)
Spain	MSW, sewage sludge	SimaPro	cradle-to-grave	1 L of sewage sludge	Gómez et al. (2010)
Sweden	Agriculture residues	OpenLCA	Gate-to-gate	1000 kg	Kimming et al. (2011)
England	MSW	SimaProv.8.3.0.0	cradle-to-gate	1 ton of kitchen waste	Tunesi (2011)
Netherland	MSW (Straw, grass, and paper)	Eco-indicator 99	cradle-to-grave	1 ton of biomass	Dornburg and Faaij (2006)
Saudi Arabia	MSW	GaBi	Gate-to-gate	1 ton of MSW	Present Study

### 4.2 Current challenges and prospects

**FIGURE 7:**
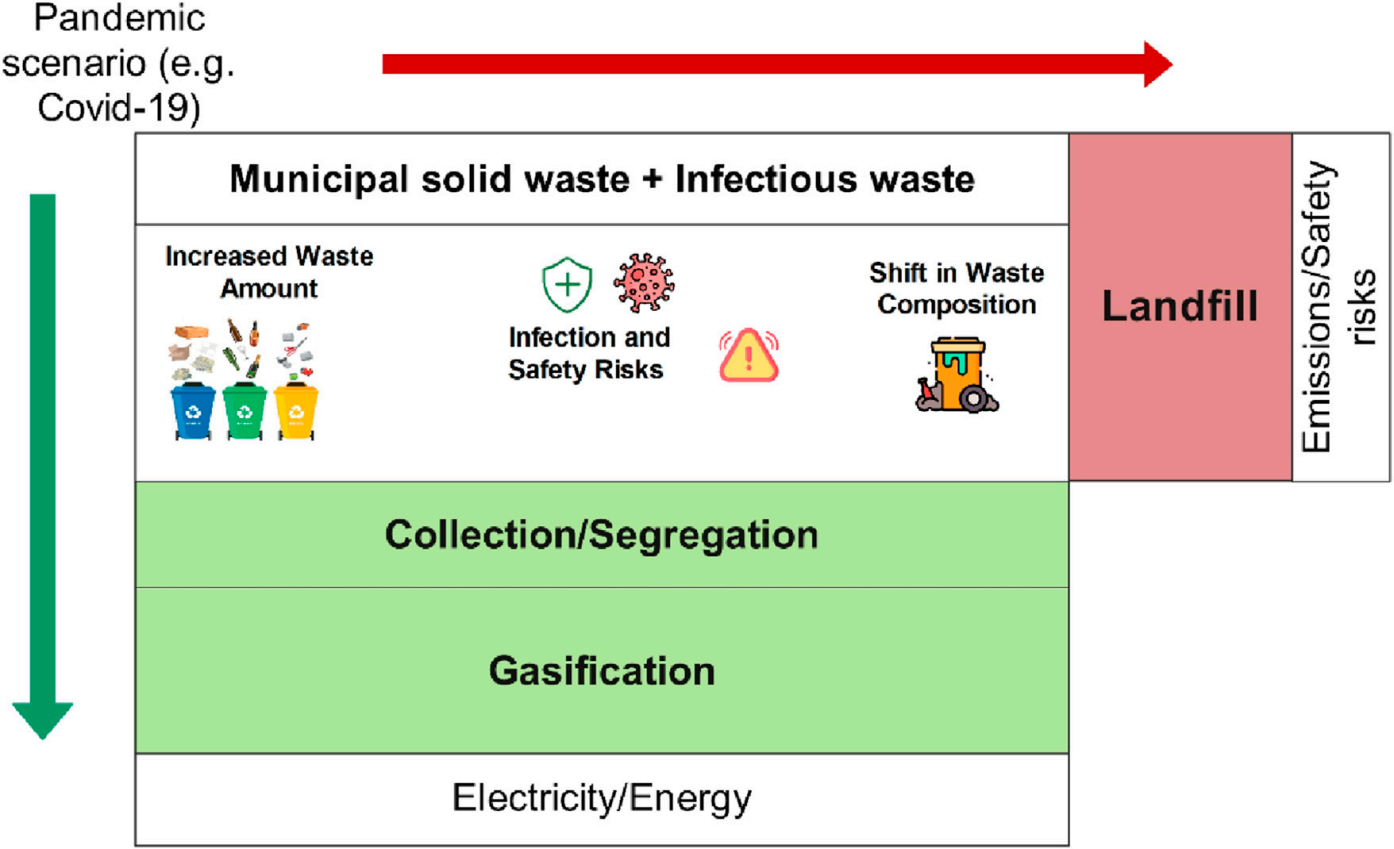
Comparison of gasification and landfill scenarios of municipal solid waste.

The LCA of syngas production from mixed MSW faced significant challenges, particularly in light of the complexities introduced during the pandemic. First, the composition of mixed MSW, which can be exacerbated by a sudden influx of challenging materials like plastic packaging and disposable items during a pandemic, poses difficulties in segregation and pretreatment. While dedicated hazardous waste requires separate handling, items like PPE and contaminated packaging often enter the MSW stream during crises, complicating management. As shown in Figure 7, infectious waste during a pandemic should be segregated and processed via gasification instead of landfilling. However, PPE, contaminated packaging, and single-use medical items require special handling to ensure proper gasification and prevent harmful emissions (Cu et al., 2023). Likewise, the absence of vigorous waste collection and management systems during pandemic surges, specifically in regions overwhelmed by healthcare demands, has highlighted logistical and monitoring gaps. Additionally, another challenge is the high energy input and capital cost required to establish syngas production facilities, which can be a barrier during economic declines triggered by pandemics (Ali and Parvin, 2022). Additionally, public awareness about proper waste separation remains low, complicating efforts to improve feedstock quality for syngas production.

Despite these challenges, the future of applying LCA to syngas production from MSW appears promising, particularly in its potential to mitigate environmental impacts during global crises such as the COVID-19 pandemic. Syngas production can play a crucial role in managing pandemic waste sustainably due to advancements in gasification technology and waste treatment processes (Nemmour et al., 2023). Transforming hazardous waste into clean energy not only reduces landfill dependence but also reduces the risk of disease transmission through inappropriate waste disposal (Oyedotun et al., 2020). Therefore, policymakers and researchers are expected to prioritize incorporating WTE technologies into countrywide waste management strategies, particularly for pandemic preparedness. Hence, future projections include the development of distributed syngas plants near areas with high waste generation, such as hospitals, which minimize transportation challenges and enable rapid waste treatment (Radwan et al., 2021).

Advancements in LCA tools, such as the GaBi software, enable more accurate calculations of environmental and economic impacts. Increased awareness and investment in sustainable solutions related to syngas production from MSW can contribute to mitigating risks associated with future pandemics (Khawaja et al., 2024). Similarly, this research could serve as a roadmap to assist policymakers in meeting the Sustainable Development Goals (SDGs), including SDG 3 (good health and wellbeing), SDG 7 (Affordable and clean energy), SDG 12 (Responsible consumption and production), and SDG 13 (Climate Action). The present study’s findings proved the feasibility of economic and environmental valuation as a win-win situation.

### 4.3 Practical implications of the study

This study demonstrates that MSW gasification has significant practical potential for producing syngas and electricity through a sustainable route. Syngas production from MSW gasification may reduce landfilling, promote sustainable waste management, and support circular economy principles (Javed et al., 2025a). It also helps to generate alternative energy, lessen dependency on fossil fuels, and help mitigate global warming (Al-Athamin et al., 2021; Sharma et al., 2020; Rabiee et al., 2024). Syngas production from MSW also creates economic benefits in local communities and a new revenue source for municipalities. This process improves energy recovery, eases waste management burdens caused by pandemics, and contributes to national energy self-sufficiency.

Economic analysis indicates strong viability; a critical examination of the by-product revenue assumptions is necessary. The optimistic market values for tar ($0.30/kg) and solid residue ($0.05/kg) are contingent upon the existence of stable local markets, which may not yet be fully developed. The payback period is most sensitive to fluctuations in electricity pricing, as a reduction to $0.07/kWh shortens the payback significantly more than other variables extend it (as shown in Figure 6). This highlights the critical importance of securing favorable electricity rates or incentives for project feasibility, whereas the project demonstrates relative resilience to increases in operational costs or decreases in by-product revenue. The sensitivity analysis performed in Section 3.6 demonstrates the project’s resilience to a significant drop in these prices, including a boundary condition where tar has no market value, which still results in a viable payback period of 5.8 years.

For the full economic potential to be realized, parallel efforts to develop these markets and establish clear regulatory frameworks for by-product utilization are essential. Future economic analyses could consider even more comprehensive risk scenarios, such as net disposal costs for tar, to provide a full spectrum of financial outcomes.Tar, a complex and potentially hazardous mixture, requires further processing or a specialized industrial user (e.g., as a fuel or chemical feedstock), which involves additional handling and potential regulatory hurdles. Similarly, the demand for solid residue is highly dependent on local construction practices and regulatory approval for its use as a secondary aggregate.

In this study, the syngas production system was modeled using a simulated fluidized bed gasifier operating within a high-temperature range (700 °C–900 °C), ensuring consistent decomposition of waste into syngas across variations in feedstock. The modeling approach demonstrates the scalability of gasification systems, including their ability to handle varying waste volumes in diverse geographic regions, particularly in rural areas lacking conventional waste infrastructure. The outcomes of this study are constructive, bridging the gap between theoretical modeling and practical application. The findings demonstrate that gasification can enhance the resilience of a city’s general waste management infrastructure, providing a reliable disposal pathway. The sustainability assessment conducted herein includes a full life cycle analysis that covers economic, technical, and environmental dimensions, providing a comprehensive framework for electricity generation from syngas. Through detailed LCA, scenario modeling, and uncertainty analysis, the study identifies and quantifies key areas for process improvement.

The gasification process requires focused investment to optimize operational parameters and reduce associated costs. The effective implementation of feedstock pre-processing and emissions control systems requires collaboration among government entities, academic institutions, and industry stakeholders. International cooperation facilitates the exchange of strategies and best practices, positioning gasification as a pivotal technology in the global transition toward sustainable WTE solutions. Lastly, practical implementation also depends on workforce training and community involvement. Educating stakeholders on the benefits of syngas production will enhance public acceptance and participation in both waste segregation and overall management efforts. By prioritizing these practical actions, syngas production through gasification can evolve from theoretical feasibility to a tangible, real-world impact, establishing a long-term, resilient, and economically viable WTE conversion strategy (Javed et al., 2025a).

### 4.4 Study assumptions and limitations

The whole study depends on modeling and simulation rather than empirical experimentation. Furthermore, while this study emphasizes the role of gasification in crisis resilience, it is essential to note that the applied gate-to-gate system boundary excludes upstream waste collection and transportation, as well as downstream electricity distribution. These stages are critical for a full resilience assessment during a crisis, as disruptions in logistics or grid stability can significantly impact the overall system’s effectiveness. Their exclusion limits the direct extrapolation of our findings to holistic pandemic preparedness, and future studies should incorporate these stages to provide a more complete evaluation of resilience.

All techno-economic and environmental outputs are projections from the model, not based on operational plant data. The gasification process, syngas yield, and subsequent electricity generation were not physically implemented. The study was modeled using primary waste data and secondary input parameters from the literature and life cycle databases (Ouedraogo et al., 2021; Al-Athamin et al., 2021). Although this permits scalable analysis and policy relevance, it excludes real-world operational uncertainties, such as variations in feedstock moisture, system degradation, and unanticipated emissions. Additionally, the assumed MSW composition and process efficiencies may not accurately capture site-specific or temporal variations in real waste streams. This study provides a simulation-based case study for the city of Jeddah and a methodological framework that could be adapted by other cities, pending further pilot-scale validation.

This study depends on the assumption of the constant composition of MSW, which varies significantly. It may not accurately reflect real-world scenarios, where variations in feedstock and operating conditions can impact syngas quality. Moreover, for the LCA inventory, this study relies on secondary data sources (Sarkodie and Owusu, 2021; Ouedraogo et al., 2021), which may lead to inconsistencies in the results. Furthermore, the economic analysis focuses primarily on syngas and electricity production, potentially overlooking valuable opportunities for by-product utilization. Additionally, the stagnant nature of the LCA may not fully capture the complexities of dynamic systems, and the lack of regional specificity may limit the applicability of the findings to diverse contexts. Future studies should confirm these modeled outcomes through experimental trials to refine assumptions and enhance the reliability of the findings.

## 5 Conclusion

This study demonstrates that the gasification of MSW offers a sustainable pathway for energy recovery while addressing critical environmental challenges. The LCA reveals significant net reductions across key impact categories, with a GWP benefit of −100 kg CO_2_ eq. per ton of waste processed, along with substantial offsets in FDP (−32.5 kg oil eq.) and FPMFP (−0.025 kg PM2.5 eq.). These results highlight the dual advantage of WTE conversion, which not only diverts waste from landfills but also displaces electricity generated from fossil fuels. Economically, the model proves viable, with an annual revenue of USD 516,474 and a 4.8-year payback period for a 50-ton-per-day facility. The system aligns with circular economy principles (SDG 12) through its potential to monetize by-products, such as tar and residues. However, the realization of this revenue is contingent upon market development and regulatory approval for these materials. Despite this uncertainty, the project’s high ROI and positive NPV, which persist even when considering scenarios with significantly reduced or zero by-product revenue, suggest strong economic potential and scalability. It underscores its strong economic potential and scalability. Notably, the USD -10.77/ton external cost savings from avoided emissions (CO_2_, NO_x_, etc.) further validate its societal benefits.

The growing frequency of urban crises underscores the urgency of building resilient infrastructure. This simulation study suggests that gasification could strengthen waste management systems by offering a potential pathway to process mixed MSW surges and generate energy during disruptions. This may help reduce public health risks associated with waste accumulation (SDG 3) and reduces landfill-induced methane emissions. However, reliance on simulated data warrants future pilot testing to address real-world variability in feedstock composition. By integrating environmental, economic, and social benefits, MSW gasification emerges as a transformative tool for sustainable development, offering a blueprint for cities navigating waste and energy transitions. Its alignment with SDGs 7, 12, and 13 makes it a compelling candidate for global decarbonization strategies.

## Data Availability

The original contributions presented in the study are included in the article/supplementary material, further inquiries can be directed to the corresponding author.
